# Shifting to Tele-Mental Health in humanitarian and crisis settings: an evaluation of Médecins Sans Frontières experience during the COVID-19 pandemic

**DOI:** 10.1186/s13031-022-00437-1

**Published:** 2022-02-14

**Authors:** Khasan Ibragimov, Miguel Palma, Gregory Keane, Janet Ousley, Madeleine Crowe, Cristina Carreño, German Casas, Clair Mills, Augusto Llosa, M. S. F. Mental Health Working Group, Ana Marques, Ana-Maria Tijerino, Anneli Droste, Carolina Echeverri, Nadja Fredj, Raghda Sleit, Sylvie Fagard Sultan

**Affiliations:** 1 Epicentre, Paris, France; 2 Médecins Sans Frontières, 34 Avenue Jean Jaurès, 75019 Paris, France; 3 Médecins Sans Frontières, Barcelona, Spain; 4 Fundación Santa Fe University Hospital, Universidad de Los Andes, Bogotá, Colombia

**Keywords:** Tele-mental health, Telepsychiatry, Tele-counselling, Remote mental health, MHPSS

## Abstract

**Background:**

‘Tele-Mental Health (MH) services,’ are an increasingly important way to expand care to underserved groups in low-resource settings. In order to continue providing psychiatric, psychotherapeutic and counselling care during COVID-19-related movement restrictions, Médecins Sans Frontières (MSF), a humanitarian medical organization, abruptly transitioned part of its MH activities across humanitarian and resource-constrained settings to remote format.

**Methods:**

From June–July of 2020, investigators used a mixed method, sequential explanatory study design to assess MSF staff perceptions of tele-MH services. Preliminary quantitative results influenced qualitative question guide design. Eighty-one quantitative online questionnaires were collected and a subset of 13 qualitative follow-up in-depth interviews occurred.

**Results:**

Respondents in 44 countries (6 geographic regions), mostly from Sub-Saharan Africa (39.5%), the Middle East and North Africa (18.5%) and Asia (13.6%) participated. Most tele-MH interventions depended on audio-only platforms (80%). 30% of respondents reported that more than half of their patients were unreachable using these interventions, usually because of poor network coverage (73.8%), a lack of communication devices (72.1%), or a lack of a private space at home (67.2%). Nearly half (47.5%) of respondents felt their staff had a decreased ability to provide comprehensive MH care using telecommunication platforms. Most respondents thought MH staff had a negative (46%) or mixed (42%) impression of remote care. Nevertheless, almost all respondents (96.7%) thought tele-MH services had some degree of usefulness, notably improved access to care (37.7%) and time efficiency (32.8%). Qualitative results outlined a myriad of challenges, notably in establishing therapeutic alliance, providing care for vulnerable populations and those inherent to the communications infrastructure.

**Conclusion:**

Tele-MH services were perceived to be a feasible alternative solution to in-person therapeutic interventions in humanitarian settings during the COVID-19 pandemic. However, they were not considered suitable for all patients in the contexts studied, especially survivors of sexual or interpersonal violence, pediatric and geriatric cases, and patients with severe MH conditions. Audio-only technologies that lacked non-verbal cues were particularly challenging and made risk assessment and emergency care more difficult. Prior to considering tele-MH services, communications infrastructure should be assessed, and comprehensive, context-specific protocols should be developed.

## Introduction

The treatment gap for mental health (MH) services in low- and middle-income countries (LMIC) is a chasm, with the ratio of LMIC MH therapists estimated at only 0.5% of that available in high-income countries [1]. When humanitarian crises (resulting from of armed conflict, natural disasters, or disease outbreaks) are superimposed onto the underlying adversity often experienced in LMIC, MH needs become even more acute, but access to care usually remains unavailable [2].

‘Tele-MH services’ (or care delivered remotely by MH care providers through telecommunications technology) are becoming an increasingly important way to expand care to underserved groups [3]. These methods have been highly effective in high-income countries [4–6] and their use and effectiveness in LMIC has been seen across diverse settings and MH conditions [7–12]. There are numerous advantages to tele-MH solutions, including reduced travel, wait times and costs, and improved access to services [13–16], especially in understaffed, remote, or insecure areas. In some cases, they are considered an enabling and empowering form of service delivery and have increased patient and caregiver satisfaction rates [17, 18]. However, implementation and use of tele-MH care in humanitarian and crisis settings remain understudied.

These interventions are by no means a panacea. Systematic reviews of digital MH solutions in 2020 and 2021 found only ‘moderate’ clinical effectiveness in LMIC and emphasized that robust study on the topic remains limited [7, 19]. Remote MH services may reinforce systemic inequalities in access to care for populations with a substantial digital divide [20]. There are also serious ethical questions about the use of tele-MH tools across settings. MH data is more sensitive, personal, and potentially stigmatizing than other health data [21]. In low-resource and humanitarian contexts, medical privacy norms may not be as firmly established or regulated, and medical ethicists worry that remote services may not protect people from substandard MH care or unwanted use of their personal information [22]. With weak evidence of tele-MH care’s efficacy over traditional, in-person therapeutic methods, these concerns must be seriously considered.

Nevertheless, during the 2020 COVID-19 lockdown, unease about tele-MH solutions had to be weighed against the threat of discontinuing care entirely for patients whose movement was restricted. Médecins Sans Frontières (MSF), a medical humanitarian organization offering MH and other care in low-resource and crisis settings around the world, assessed this risk carefully before changing a substantial part of its MH activities to remote care in April of 2020. The shift was rapid and necessary, affecting virtually every type of MSF MH program and therapeutic intervention (more detail in the methods and Tables 1 and 2 and Fig. 2), but allowed minimal preparation in many cases. Some facilities had little technological infrastructure or no previous experience using tele-MH care. Others had staff or patients who were skeptical of this change. As a result, the organization decided to concurrently evaluate the altered service provision in an effort to understand the challenges and successes of implementing tele-MH care in a tumultuous period, and to better prepare for similar remote care needs in the future. To our knowledge, this is the first assessment of MH staff perceptions of tele-MH care during the COVID-19 pandemic in humanitarian, crisis, or otherwise low-resource settings.

**Table 1:** MH patient type served by sampled MSF Mental Health Care Activity Managers (N = 81)

Patient type served by MSF Mental Health Program	n	%
Primary Health Care	42	51.9
Sexual violence survivor	42	51.9
Migrants (internally displaced, refugees, other)	36	44.4
COVID-19 suspects/patients	35	43.2
Urban population (chronic violence settings or lack of care)	26	32.1
Long-term chronic disease/adherence support (HIV, TB, NCDs)	19	23.5
Malnutrition program participant	15	18.5
Maternal Health Program	12	14.8
Surgical/burns patients	8	9.9
Non-COVID-19 outbreak support	2	2.5
Pediatric patients	1	1.2

Data derived from multiple choice question where respondents could select more than one option

**Table 2:** Inclusion criteria in projects with MH component by age and gender (n = 81)

Inclusion criteria by age		Inclusion criteria by gender	
All age groups	56 (69.1%)	Any gender	74 (91.3%)
Except children or/and elderly	16 (19.7%)	Male only	6 (7.4%)
Only either adult, elderly or children	9 (11.1%)	Female only	1 (1.2%)

## Methods

This study was undertaken in June-July 2020 using a mixed method, sequential explanatory design [23]. Staff from projects providing any type of MH intervention were invited to participate regardless of the extent or existence of remote service provision in their project site. Participation was restricted to those in managerial or supervising roles with a breadth of information about MH care provision across their facilities, though these staff were not always patient-facing themselves. For simplicity’s sake, these are henceforth referred to as “activity managers”.

### Quantitative data collection

An online questionnaire was based on core themes identified in the literature as well as discussions with the research team (that included both MH and epidemiological experts). Tele-MH service provision was explored with activity managers from sites that shifted to remote care and those that were not able to, the former answering more detailed questions about feasibility, perceived acceptance and performance. 120 activity managers were invited to participate in an online survey through their official MSF email address, of which 84 (70.0%) provided consent and 81 (67.5%) participated. After completing an online informed consent process, a link to the quantitative questionnaire was sent to the participants. Quantitative data was entered and analysed using R v.3.6.5.

### Qualitative data collection

To explore quantitative findings in more detail in-depth interviews (IDI) were conducted subsequently. IDI scripts were developed based on themes identified during questionnaire development and according to preliminary quantitative results. Data were collected until the point of saturation when no new themes emerged from interviews. Investigators sought a sample with maximal heterogeneity to capture as wide a spectrum of experience as possible, and therefore staff from every region where MSF has MH operations were included. The sample ultimately included staff from a variety of humanitarian contexts, and only two of 13 IDIs were from sites that did not shift to remote care. Interviews were conducted through audio or audio-visual equipment in English (except for one participant who indicated a preference for French, and whose interview was subject to English translation prior to analysis) and could be paused or ended at any time if requested by the participant.

Confidentiality was maintained by not including participant names or defining characteristics throughout the transcription. IDI transcripts were reviewed for quality and errors by the interviewer before being uploaded to NVivo software v1.3. Authors then independently open-coded the true-speech, verbatim transcripts for themes and developed and refined an inductive, thematic structure. Descriptive and content analysis of transcripts was conducted.

### Ethics

This study was approved by MSF internal Ethical Review Board (Protocol ID: 2028). Inclusion was voluntary and required written informed consent. Participants provided permission for use of audio recordings and verbatim quotation. Datasets used in the study are available upon reasonable request to MSF-France’s Medical Director via the study’s corresponding author.

## Results

### Quantitative survey

**Fig. 1:**
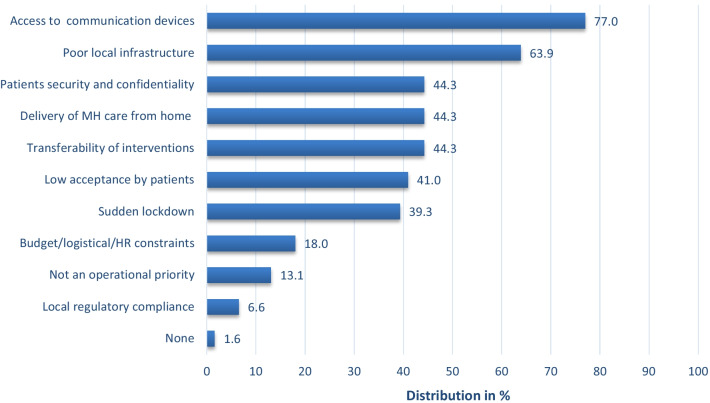
Perceived barriers to the use of remote MH services among MSF projects that transitioned to tele-MH care (n = 61)

**Fig. 2:**
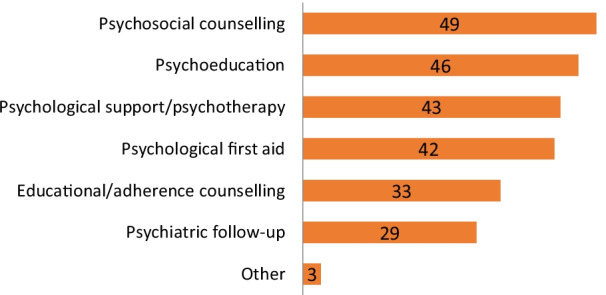
MH interventions switched to remote format (n = 61)

81 online surveys were completed (67.5% response rate) from activity managers located in 44 countries and 6 world regions. Most respondents were in Sub-Saharan Africa (39.5%), the Middle East and North Africa (18.5%), and Asia (13.6%). Programs provided care for every MH condition regardless of severity, as per MSF policy (even if in some circumstances reliance on external referrals remain essential to expand the scope of interventions) and most projects represented in the sample had no restrictions in terms of age (69.1%) or gender (91.3%) (Table 2). Tele-MH implementation held complex challenges (Fig. 1), but 61 (75.3%) of all participants managed to overcome these barriers and initiate a transition to remote care, despite providing a wide variety of MH interventions (Fig. 2). Notably, most tele-MH interventions depended on audio-only platforms (80%)—phone or voice chat software—with video consultation available for only 20% of projects.

Among the projects that implemented tele-MH services (n = 61), nearly a third (30%) of activity managers reported that more than half of their patients could not be reached using this format of care. Poor network coverage (73.8%), a lack of communication devices (72.1%), or a lack of a private space at home (67.2%) were the leading obstacles to remote care. Nearly half of activity managers (42.6%) thought children were most often excluded when tele-MH services were exclusively used, while nearly a third (29.5% and 31.1% respectively) thought this of the elderly and people with severe MH conditions. Thirty-three participants reported that there were at least some patients showing increased engagement with tele-MH care, and 36.4% reported that adults engaged better than other age groups, with no gender difference. Half (54%) of the respondents reported that patients indicated concerns about the privacy of their tele-consultations, with a lack of a private space for confidential conversations cited as a primary worry. A notable 42.4% of respondents perceived this as linked to fear of sexual or interpersonal violence. Other reported concerns were stigma (6.1%) and a lack of trust in MSF (3%). Language barriers, the difficulties of incorporating interpreters into remote care models, missed appointments or scheduling conflicts, a lack of non-verbal cues during audio-only interviews, and a lack of clear protocols and guidelines were also cited as barriers to successful tele-MH care.

**Fig. 3:**
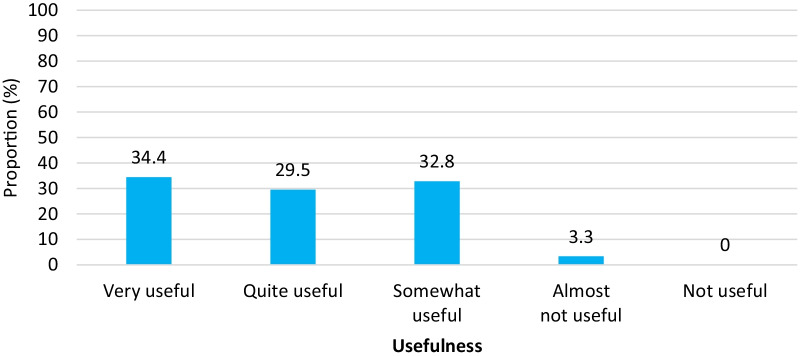
Perceived usefulness of tele-MH care as an alternative to in-person sessions (N = 61)

Activity managers indicated that their staff had variable capacity to conduct important patient assessments, with 21.3% of managers reporting some staff unable to remotely conduct a full MH assessment, and 31.3% reporting some staff unable to conduct a protection risk assessment. Nearly half (47.5%) of respondents felt their staff had a decreased ability to provide comprehensive MH care using telecommunication platforms, with some reporting a similar (19.7%) or increased (3.3%) ability to provide comprehensive care. When asked to rate their project’s perception of the effectiveness of tele-MH services compared to in-person sessions, most thought staff had a negative (46%) or mixed (42%) impression of remote care. The most common tele-MH care needs cited by managers were training (41.7%) (including on patient assessment and management), clear guidelines and protocols (22.9%), and communication devices (12.5%) for both patients and staff. Nevertheless, despite the challenges, almost all activity managers (96.7%) thought tele-MH services had some degree of usefulness as an alternative to in-person consultations (Fig. 3), most commonly citing improved access to care (37.7%) and greater time efficiency (32.8%) as reasons for its continued use.

### Qualitative assessment

Following the completion of the online survey, 48 participants consented to IDI on the topic. After interviews with the first 13 respondents, investigators believed that a point of saturation had been achieved because no new themes were emerging, so further IDIs were discontinued. IDI participants represented 6 geographic regions as well as multiple MH program types (migration and forced displacement, chronic conflict or violence, natural disasters, post-conflict contexts, etc.).

### Advantages of tele-MH care

Overall, activity managers interviewed felt grateful that remote care options were available during the pandemic, though all still expressed a preference for in-person care. They described greater accessibility for some patients, time efficiency (more consultations, better time management), and reduction in travel-related costs for both patients and MH care providers.

“[Counsellors can] stay in the office and call every patient, every week quite easily! That is why I think it is time efficient, because [before] we spent so much time on the road”

“I would always recommend tele-counselling to most MH programs because they are an option for people who cannot come to the facility… [and] offer…services regardless of the day and time.”

Notably, prior to providing tele-MH services in their facilities, half of the IDI participants described thinking it would not be feasible to do so in their clinical setting assuming these services would have low acceptance by patients, that there would not be enough communication devices, internet, phone network, or call credit, and that their team’s organization and abilities would be insufficient for the task. These interviewees described being positively surprised by the experience of being forced to provide remote care, with one manager stating.

“Now, everything we thought has changed because…we realized that [tele-MH services] will be important…and they worked; the teleconsultations really had a positive impact.”

#### Challenges providing tele-MH care

“Some patients cannot do in-person sessions. In this case, tele-counselling really helps a lot. Other than this, there are mostly disadvantages…”

Despite some advantages, the challenges surrounding the sudden transition to tele-MH services were numerous. Some types of remote sessions were considered particularly challenging, especially psychiatric assessments of new patients and counselling prior to initiating a long-term medical treatment. More routine MH services (such as adherence or psychosocial counselling, psychological care, psychiatric care, etc.) were described by one respondent as “difficult…but doable”.

#### Communications infrastructure

Respondents described the many device-related challenges they faced when transitioning to tele-MH care: indigent patients often lacked phones, tablets, computers, internet access, cell phone signal, and money or credit necessary for a tele-consultation. Most MSF staff provided remote MH care using nothing more than basic, non-video-enabled mobile phones. Group therapy sessions with participants in multiple locations were not possible. When automated billing plans were unavailable (as is common in many LMIC), both the patient and provider’s phone would have to be periodically ‘filled’ with pre-paid credit. IDI respondents described the stress associated with remote sessions, especially because they often ran longer than in-person consultations or risked being interrupted due to insufficient phone credit. Moreover, mobile phone network coverage was often dependent on location and time, particularly in rural sites and during peak hours, which also led to dropped calls, background or other noise, and other connection difficulties that could be particularly stressful when patients were at high-risk of serious MH sequelae or other harm.

Additionally, many of MSF’s diverse settings depend on language translators as part of the therapeutic environment. This proved difficult to replicate virtually, with complex three-way calling procedures in some mobile networks, network and connection challenges, confidentiality issues, and a lack of available translators overall during the pandemic period.

#### Excluded and vulnerable populations

Many patients did not own a communication device and thus could not access care without sharing or borrowing someone else’s. This was particularly the case for female, pediatric, and geriatric MH patients and, in some cases, potentially left some individuals more vulnerable to controlling or abusive family members during the period of remote MH care. However, phones provided by MSF could also trigger issues of privacy and household power dynamics that could threaten the robustness of MH care provision. Participants reported that abusive individuals (husbands, parents, etc.), who would usually be excluded from the consultation room prior to COVID-19, tended to normalize a patient’s suffering, intensify MH stigma, and sometimes impede contact with MH professionals.

*“When we are talking about children, the family members say: ‘he or she is okay’ …But it doesn't mean that the child is exactly okay. Maybe a child is suffering, maybe being abused…we don't know. And they will not tell us because if we can reach [the children], the family is going to be around...So how can we know what is happening to a child in this context?”*

*“It is not nice for some women to receive a call [from a male counsellor]. Sometimes this can be an issue because we have only three women counsellors...Women often don’t want to share their problems due to fear or their family members’ negative attitudes (often a husband or a mother-in-law). Therefore, they refuse to receive help.”*

IDI participants described how pediatric and female patients would sometimes participate in consultations with other family members present, either because a household member wanted to actively monitor the session (out of fear that the patient would disclose certain information) or because the reality of their living situation prevented private communication. In these cases, the remote therapeutic process was seriously hindered.

*“Even if we get consent from the parents [of a pediatric patient], it is not possible for children to have a whole room to themselves. They can misspeak because the parents are scared that a child will express something negative about the family…. For women, this is when there are scenarios related to domestic violence…”*

Vulnerable populations also included those with underlying risks that need to be assessed and managed (violence survivors, attempted suicide, etc.), a task that was particularly difficult remotely. This was enough of a concern that four MSF projects providing emergency MH services continued in-person care despite COVID-19 considerations.

#### The therapeutic alliance

*“…when you have the patient face to face, you can tell by their non-verbal language, what is going on with the patient... So, it helps a lot, it helps a lot to see consistency in what the patient is saying and what you are actually observing”*

The challenges of MH care provision over the phone or through a screen were described by most respondents. Audio-only remote consultations, a reality for most MSF settings, were often a serious limitation, and managers described some staff even using their own personal smartphones or computers to provide remote video consultations. However, not all felt that way; a few participants described audio-only sessions as advantageous with populations or care providers who may not have been as accustomed to interacting through a screen:

*“Some people are not comfortable on camera…because it is a different connection. Like I'm talking to an image—not to a person—and the environment is different. I need to feel comfortable when talking with someone because my own voice, tone, and image are different. We don't have [these things] when we are at an [in-person] session. You go, you see the patient, and we talk.”*

### Staff needs and recommendations

Nearly half of interviewees described the difficulty related to not having protocols or guidelines to advise them on tele-MH care. Some described hastily adapting other groups’ protocols to fit their patients. Scheduling conflicts and difficulty managing remote appointments, especially with new patients, missed appointments, long sessions, and overtime work for staff (due to increased caseloads, 24/7 availability, and increased session lengths and frequency) were all unexpected challenges that managers felt they could be better prepared for in the future. Most managers were not keen to continue the new remote care models except for “short periods” or for specific patients, especially those who travel long distances to care. Some managers also advised that, moving forward, supervisors should pay better attention to the emotional needs of their team members and encourage teamwork.

## Discussion

Although the feasibility and effectiveness of tele-MH care provision in low-resource settings has been investigated, none to date has looked at its use in humanitarian environments where patients may have been exposed to conflict, violence, forced migration, or other extreme conditions [3, 7]. In these contexts, tele-MH solutions provided a lifeline to many patients who would have otherwise been entirely cut off from care during the pandemic. However, these data also seem to support prior research showing that the most marginalized and vulnerable patients (those who have the least economic means or education, are very old, those that need a translator to speak with their care provider, have severe MH conditions, or have survived abuse) are often not reached by these services or are less engaged when reached [24]. Children were another vulnerable group who struggled to access teleconsultations in our study, though this finding departs from other published research [25, 26].

In situations of abuse, our results even seem to suggest that remote care may place some patients at higher risk of violence when household members have access to privileged conversations or feel threatened by a patient’s relationship with their therapist. Indeed, for patients such as survivors of sexual and interpersonal violence, a cautious and non-intrusive approach should remain standard, as should the use of “code” and “safety” words. Potential risks must be the object of constant regard, while patients remain in full control of the sessions’ content and duration [27]. It should be noted that for some patients, remote solutions may never be the best fit, and clinicians should not hesitate to encourage an in-person approach if other risks can be managed, as was the case in several MSF MH projects even during the most restricted COVID-19 periods.

It is notable that so many of our respondents doubted the efficacy of tele-MH approaches prior to the pandemic, despite published evidence of effectiveness [7–13]. Indeed, some staff were openly resistant to change while others struggled to adjust to the new format of care. This can be partially explained by the fact that MH services in humanitarian settings are generally provided by non-specialist clinicians trained in task-shifting approaches. These clinicians may have less experience, ability to engage effectively with patients, or understanding of how to translate their work to remote models of care that require sophisticated verbal communication, transcultural, and behavioral skills [28]. Yet, most activity managers were pleasantly surprised at their facilities’ ability to transition to these platforms and viewed tele-MH as a satisfactory alternative in the pandemic, while still expressing preference for face-to-face sessions.

Thus, even in places with no prior experience using technology to deliver care, and even when substantial infrastructure challenges were present, remotely delivered therapy was possible for some patients, especially hard-to-reach groups. Moreover, participants found it time and cost-effective, mostly by alleviating travel-related costs. These considerations are particularly important for patients who need to isolate, are migrants, are geographically dispersed, need a clinician with a similar cultural background (such as in refugee settings), or are in places that are often inaccessible to experienced therapists (conflict-affected countries, detention centres, etc.).

However, a note of caution should also be added: the infrastructure challenges described in these settings were onerous. Communications networks are often under-developed in low resource settings and absent or severely disrupted during periods of crisis. This was the case in most of our settings, and it is possible that MSF teams’ negative impressions of remote care were partly due to the audio-only sessions that were often their only option. Participants mentioned a lack of non-verbal cues and body language and its impact on patient-practitioner interactions as a key challenge to successful tele-sessions. Preference for video platforms was also displayed by therapists who opted to use their own personal smartphones when patients had devices with a camera, rather than relying on their MSF-provided audio-only device. In fact, previous research has shown that higher sound and video picture quality are associated with greater tele-psychiatry patient acceptance, engagement, and satisfaction [24, 25]. Moreover, poor network connection was distressing in circumstances that involved sensitive conversations or high risk for patients. Thus, a firm understanding of technical capabilities prior to implementing digital MH solutions is needed, as are quality devices and strong connectivity for both patients and providers. Appropriate staff supervision and support, evidence-based guidance and tools, and programs adapted to humanitarian contexts are also critical for future projects [29].

## Limitations

Our results are limited by the composition of our sample. We were only able to survey and conduct interviews with MH staff, leaving the patient perspective unknown. Future research should capture their satisfaction with tele-MH services in these settings. Additionally, all respondents were active MSF staff at the time of their participation and were contacted through their official work email address, though scrupulous efforts were made to assure confidentiality and investigators’ neutrality. Participants’ gender and other demographic variables were not collected, so we are unable to say how these may or may not have influenced results, though they likely did in meaningful and potentially significant ways. Moreover, an online survey response rate of two thirds—likely driven by extreme time constraints of activity managers amid a pandemic crisis—may limit the generalizability of findings to other contexts, especially in the absence of data to allow comparison between respondents vs. non-respondents. The interviewed sample is likely representative of the range of projects in MSF portfolio, since point of saturation was reached at 13^th^ IDI (out of 48 consenting participants), but it does likely underrepresent those less comfortable in English. Respondents were all managers/supervisors, meaning that their answers may have been incomplete or distorted by their own biases, may have been more complimentary (or critical) of their own staff or projects, or may have overlooked or misunderstood power dynamics affecting their project.

## Conclusion

Among these staff in low-resource, humanitarian settings, tele-MH activities were an acceptable and sometimes superior alternative to in-person therapeutic interventions during the COVID-19 pandemic lockdowns in 2020. However, they were not considered suitable for all patients, and have the potential to exclude and expose some patients to additional risk, especially survivors of sexual or interpersonal violence, pediatric and geriatric cases, and patients with severe MH conditions. Audio-only technologies that lacked non-verbal cues were particularly challenging and made risk assessment and emergency care more difficult. Prior to implementing tele-MH services, communications infrastructure should be assessed, and comprehensive and context-specific protocols (including clinical training and supervision guidance) should be developed.

## Data Availability

The datasets used in the study are available upon reasonable request to MSF-France’s Medical Director via the study’s corresponding author.

## Appendix 1

### Questionnaire about the implementation of tele-MH activities

MSF has had to rapidly adapt its activities to respond to COVID-19 pandemic. The need for physical distancing and other movement restrictions has led, in some programs, to the sudden transition to remote models of delivering care. In Mental Health (MH) programs, solutions like tele-MH have been considered or implemented.

We define ‘tele-MH’ here as any type of MH intervention performed by a MH care provider remotely over a technological device (phone, smartphone, tablet, computer). This can range from punctual interventions like psychoeducation or Psychological First Aid (PFA) through crisis hotlines to the regular individual or group counselling, psychotherapy, or psychiatric follow-up. Other activities provided remotely like case supervision or multidisciplinary team meetings are not considered, in this evaluation, as part of tele-MH. Furthermore, we will restrict the scope of tele-MH to interventions aimed at MSF beneficiaries (and not as part of staff health care).

The sudden implementation of a functioning tele-MH system in humanitarian contexts involves characteristics that are worth exploring. With the present questionnaire, we aim to identify challenges and lessons learned during the process of implementation of tele-MH (either in context of the switch from face-to-face care or primary implementation), so that MSF (and other humanitarian agencies) can better support staff and assist the transition in all its fields. Therefore, this questionnaire is to be answered by a person most closely involved in this process of transition.

As a key person who supervised the implementation of the tele-MH, we kindly request your collaboration to participate in our study by filling in the questionnaire below, which might take around 10–20 min to complete.

Thank you very much for your collaboration!

______________________________________

Date:

Mission country:

Project:

Role: ☐ MHAM / ☐ PSAM / ☐ MH Supervisor / ☐ MTL or MedRef / ☐ Other:

**V. Project characterization**

1. Which type(s) of programs with MH activities are present in your project? Please choose all that apply.
  - ☐ Migrants (refugees/asylum-seekers/IDPs/people on the move) program
  - ☐ MHPS program in primary health care
  - ☐ Sexual violence program
  - ☐ Urban violence and excluded populations (e.g., sex workers, people who use drugs, street families, survivors of torture and ill-treatment, detainees…)
  - ☐ Surgical/burns program—liaison
  - ☐ Maternal health program—liaison
  - ☐ Nutrition program
  - ☐ Long-term chronic diseases (HIV, TB, NCD) program
  - ☐ Non-COVID-19 outbreak (e.g., Ebola, Marburg virus disease, Lassa fever…)
  - ☐ New COVID-19 related program
  - ☐ Other:
2. Which age groups are part of the inclusion criteria of the project? Please choose all that apply.
  - ☐ Children
  - ☐ Adolescents
  - ☐ Adults
  - ☐ Elderly
3. Which genders are part of the inclusion criteria of the project?

- ☐ Female
- ☐ Male
- ☐ Another gender category (or categories):
- ☐ Any gender

**VI. Implementation feasibility**

1. Were you able to implement tele-MH in the project?
  - ☐ Yes (even if the process is not completely finalized)
  - ☐ Partially (was not possible in some programs)
  - ☐ No

If “Partially”, please specify programs in which tele-MH implementation was not possible?

2. Whether or not tele-MH was implemented, what were the challenges your project faced? Please choose all that apply. There is also room for free text entry at the end of this question.

RELATED TO THE LOCAL CONTEXT:

- ☐ Poor local infrastructure (network coverage, concerns about network safety/privacy…)
- ☐ Local regulatory compliance requirements (e.g., national laws or policies on data protection…)

RELATED TO THE PROJECT/OPERATIONS:

- ☐ Project budget/logistical/HR constraints
- ☐ Challenges related to sudden lockdown (not possible to collect patients phone numbers, not possible to access files, not possible to do phone distribution as planned…)
- ☐ Not considered to be an operational priority by the project (even if you disagree with the decision)
- ☐ Concerns about transferability of face-to-face interventions to remote mode (related to the content of the intervention or clinicians' skills)
- ☐ Challenges associated to the delivery of care from MH care professional’s home (e.g., ensuring that confidentiality is maintained; compromise between domestic and professional roles at home; decreased availability due to conflicting duties—children under care…)

RELATED TO PATIENTS:

- ☐ Patients access to phones/other communication devices or airtime
- ☐ Perceived low acceptance of tele-MH by patients (including cultural or gender issues)
- ☐ Concerns that tele-MH could expose patients to harm (due to lack of privacy…)

OTHER/additional information:

- ☐ Other:

**If in question B1 you chose “No”, the questionnaire ends here. Thank you for your participation.**

**If in question B1 you chose ‘Yes’ or ‘Partially’, please continue with the questionnaire.**

**The following questions refer to the program(s) where tele-MH implementation** **was possible** **(even if the process is not completely finalized).**

**VII. Process**

1. Was the tele-MH implementation considered in reaction to COVID-19 pandemic?
  - ☐ Yes
  - ☐ No
2. What are the primary goals of the implementation of tele-MH? Please choose all that apply.
  - ☐ Continuity of MH care for patients already under follow-up
  - ☐ Initiation of MH care for new patients
  - ☐ MH crisis hotline for patients already under follow-up
  - ☐ MH crisis hotline for new patients
  - ☐ Other:
3. Which type of interventions has been adapted to remote care? Please choose all that apply.
  - ☐ Psychological first aid
  - ☐ Psychoeducation
  - ☐ Educational/adherence counselling
  - ☐ Psychosocial counselling
  - ☐ Psychological support/psychotherapy
  - ☐ Psychiatric follow-up
  - ☐ Other:
4. What type of system of tele-MH is being used? Please choose all that apply.
  - ☐ Audio only
    - Please specify platform ___________________
  - ☐ Audio + Video
    - Please specify platform____________________
  - ☐ Other (please specify format):
5. Please choose the three most time-consuming steps during the implementation of tele-MH.
  - ☐ Logistical implementation of tele-MH infrastructure
  - ☐ Promotion of the new teleservices within the community
  - ☐ Training to staff
  - ☐ Protocol drafting
  - ☐ Data collection (including the adaptation of clinical files and database)
  - ☐ Reorganization of team accountabilities and internal referral system
  - ☐ Reorganization of external referral system
  - ☐ Other:

**VIII. Access and acceptance by patients**

**The following questions refer to patients that** **are unable** **to access tele-MH in programs where implementation was possible/is under way:**

1. Which proportion of the regular MH cohort(s) is unable to access tele-MH?
  - ☐ Less than 25%
  - ☐ Between 25 and 50%
  - ☐ Between 50 and 75%
  - ☐ More than 75%
  - ☐ Difficult to say
2. What are the main barriers faced by patients that cannot access tele-MH? Please choose all that apply.
  - ☐ Lack of phones/tablets/computer
  - ☐ Associated costs
  - ☐ Poor phone/internet network coverage in the area they live
  - ☐ Limited technological skills
  - ☐ Expressed concerns about the security and confidentiality of the network/intervention
  - ☐ Lack of safe space at home to receive calls for tele-MH
  - ☐ Preference of patient or caretaker
  - ☐ Other:
3. In the project, which particular subgroups are excluded from care with tele-MH solution? Please choose all that apply.
  - ☐ Men
  - ☐ Women
  - ☐ Children
  - ☐ Adolescents
  - ☐ Adults
  - ☐ Elderly
  - ☐ People with severe MH conditions
  - ☐ People with mild MH conditions
  - ☐ Any other segment of the cohort:
  - ☐ None
  - ☐ Difficult to say

**The following questions refer to patients who have access to tele-MH in programs where implementation was possible/is under way:**

Overall, do MH care providers in your team perceive any change in how involved patients are during the session or their attendance to consultations since tele-MH was implemented?

- ☐ No change at all
- ☐ Overall reduced engagement
- ☐ Overall increased engagement
- ☐ Changes in both directions
- ☐ Difficult to say/I don’t know

**If in question D4 above you chose ‘Overall increased engagement’ or ‘Changes in both directions’, please continue with the questionnaire.**

**If in question D4 you chose ‘Overall reduced engagement’ please go to question D6.**

**If in question D4 you chose ‘Difficult to say/I don’t know’, please go to question D7.**

In your project which subgroups of the cohort showed increased engagement during the session or adherence to consultations with tele-MH (compared to face to face)?

- ☐ Men
- ☐ Women
- ☐ Children
- ☐ Adolescents
- ☐ Adults
- ☐ Elderly
- ☐ People with severe MH conditions
- ☐ People with mild MH conditions
- ☐ Any other segment of the cohort:
- ☐ None
- ☐ Difficult to say

**If in question D4 you chose ‘Overall increased engagement’ please go to question D7.**

6. Which subgroups of the cohort showed reduced engagement during the session or adherence to tele-MH compared to face-to-face consultations?
  - ☐ Men
  - ☐ Women
  - ☐ Children
  - ☐ Adolescents
  - ☐ Adults
  - ☐ Elderly
  - ☐ People with severe MH conditions
  - ☐ People with mild MH conditions
  - ☐ Any other segment of the cohort:
  - ☐ None
  - ☐ Difficult to say
7. Do patients indicate concerns about privacy in tele-MH?
  - ☐ Yes
  - ☐ No
  - ☐ I don’t know
    If yes, please specify:
8. Please mention other challenges/acceptance issues patients have been facing:

**IX. Acceptance by MH care providers in your project**

1. Do MH care providers in your team feel their current skillset is appropriate for tele-MH?
  - ☐ Yes
  - ☐ Mixed (some do, some don’t)
  - ☐ No (due to their skills with technology)
  - ☐ No (due to their clinical skills)
  - ☐ No (both technological and clinical skills)
  - ☐ Difficult to say
2. To which extent are MH care providers in your team able to do a proper full mental health assessment (including the risk of harm to self and others) in tele-MH?
  - ☐ Not able at all
  - ☐ More or less
  - ☐ Overall very able
  - ☐ Mixed (some are, some are not)
  - ☐ Difficult to say
3. To which extent are MH care providers in your team able to do a proper assessment of protection-related risks (sexual violence, intimate partner violence, child safety) in tele-MH?
  - ☐ Not able at all
  - ☐ More or less
  - ☐ Overall very able
  - ☐ Mixed (some are, some are not)
  - ☐ Difficult to say
4. How do mental health care providers in your team perceive the effectiveness of tele-MH compared to face-to-face counselling?
  - ☐ Lower
  - ☐ About the same
  - ☐ Higher
  - ☐ Mixed opinions (some say lower, some say higher)
  - ☐ Difficult to say
5. Please mention other challenges/acceptance issues MH care providers in your team have been facing:

**X. Performance of tele-MH**

1. In light of the current constraints on the face-to-face consultations, how useful do you find tele-MH as an alternative solution?
  - ☐ Not useful
  - ☐ Almost not useful
  - ☐ Somewhat useful
  - ☐ Quite useful
  - ☐ Very useful
2. Compared to face-to-face consultations, to what extent are you able to provide comprehensive care (including referrals to other organizations, multidisciplinary case discussions…)?
  - ☐ Decreased ability, but only due to factors external to tele-MH (e.g., movement restrictions, reduction of services provided in the community…)
  - ☐ Decreased ability, including factors associated with tele-MH
  - ☐ Same ability
  - ☐ Increased ability

**XI. Advantages of tele-MH compared to face to face.**

1. Have you found any advantages of tele-MH compared to face-to-face sessions? Please choose all that apply.
  - ☐ Greater engagement
  - ☐ Greater time efficiency (e.g., can do more consultations or achieve more in the consultation)
  - ☐ Cheaper (for the project)
  - ☐ Improved access
  - ☐ Greater patient satisfaction
  - ☐ Greater MH care providers satisfaction
  - ☐ Other:

**XII. Recommendations:**

1. Any other recommendations:
  Click here to enter text.
2. Would you like to participate in an in-depth interview about your experiences in the implementation of tele-MH?
  - ☐ Yes
  - ☐ No
  If yes for H3, “Please provide your contact information”
  - First name:
  - Family name:
  - E-mail address:
  Thank you for your participation!

## Appendix 2

**In depth interview script**

### Experiences and challenges adapting MSF mental health activities due to COVID-19

This interview script provides an outline of questions to ask after written consent has been received from the participant. The questions will be tailored based on responses given in the online questionnaires; text in brackets with a letter and number (A1) correspond to the questions found in the online questionnaire. Examples of probing questions are given and should be asked in the case that the details do not come naturally to the participant when giving their response. They are also included as prompts for the interviewer, as are examples that are given.

The online questionnaire will be filled based on all programmes with a MH component within the project (e.g.: migrants, sexual violence, HIV…). Following, IDIs will focus mostly on the programme in which the tele-MH implementation was most relevant as an experience and will explore the challenges associated with the transition to remote mode across **different interventions within this programme**.

Examples of MH interventions include:

1. Psychological first aid
2. Psychoeducation
3. Educational/adherence counselling
4. Psychosocial counselling
5. Psychological support/psychotherapy
6. Psychiatric follow-up

Part 1—Introduction

These introductory questions are designed to build rapport with the respondent, whilst also gaining valuable background information.

Thank you for agreeing to speak with me today. As I explained before, we are interested in hearing about your **experiences and challenges in adapting mental health interventions** **to remote format** in response to the current COVID-19 pandemic. By intervention, I mean the type of MH service provided; for example, psychological first aid, psychoeducation, or adherence counselling. We are interested in learning from both the **interventions** which you were **able to adapt to** **tele-MH**, but also those that were **not able to be adapted**. We are also keen on understanding the perspectives from the point of view of **MH providers, the patient, and your own perspective** about the performance of tele-MH services. We are also interested and are aiming to interview MH providers and patients directly, however, this very much depends on time and resource availability.

Do you understand what I mean by tele-MH? Or would you like me to read our definition?

Do you understand what I mean by “intervention”?

During this interview, I will be jumping back and forth and expanding on the answers that you provided in your online questionnaire (so that you don’t have to provide us with the same information twice!).

Don’t forget that you have the right to skip any question that you feel uncomfortable with or prefer not to answer.

**Table:**

•	Can you tell me a bit about the project that you work in?
**Probes**:	How long have you been in this role for?
	Can you describe to me the programmes with a MH component?
	Who is the target population? What “intervention” types are used?
	Out of these, in which were you able to implement tele-MH?
**Probes**:	If tele-MH has been implemented, how long has it been in place for?
•	Have you used tele-MH as a tool for MH programmes previously?
**IF YES, probes**: As a manager/MH provider. What were your experiences? What was the context?	
•	When the decision to implement tele-MH was considered, how feasible did you think it would be **in your specific context**?
**Probes**:	Have your views changed since then?
	In general, what about your programme made it particularly easy/hard to implement?

Part 2—Identifying challenges

**Table:**

1.	In the questionnaire, you identified (B2) as challenges. If you had to rank these challenges in order of "hardest” to “easiest” to overcome, what would come first?
**Probes**:	Including those that you were not able to overcome. What about the second? Third?

**For this interview, I would like to focus on the xxx programme, and its corresponding mental health interventions.**

Part 3—Experiences where tele-MH implementation was possible

Now I would like to talk about your experiences where the switch to tele-MH was possible (refer to answer to introduction question 1 of IDI/ C3 in the questionnaire). I am interested in the experiences associated with these different interventions.

**Table:**

1.	I would like to go into a bit more depth with the challenges you identified. Can you expand on these for me?
**Probes**:	Were there specific challenges which were associated with each “intervention” type?
	Do you think these challenges are specific to your project/context/population?
	Are there any other challenges not mentioned?
1.	How did you manage to adapt the mental health interventions, given the challenges?
**Probes**:	Did this differ by subpopulation/intervention type, or was the same approach taken for all?
	Do you feel you were given the correct tools to overcome these challenges? What could have helped you?
	Were new interventions proposed in light of these challenges?
2.	Are there any ongoing challenges?
**Probes**: or mainly “start-up” challenges? Have you been able to mitigate the impact of these challenges?	

**Part 4—Perspectives**

3. **Access/acceptance by patients**

**Table:**

(A)	Overall, how do you think patients have felt/responded to the implementation/switch?
**Probes**:	Do the main barriers to access you identified (D2) vary by subpopulation?
	Have you noticed some **interventions** where more issues/concerns are expressed than others? How would you explain this?
	Have you noticed some **subpopulations** where more issues/concerns are expressed than others? How would you explain this?
(B)	You noted that (D3) are excluded from care. Can you explain this choice to me?
**Probes**: Are there any given subgroups (e.g., of children) that are excluded? How would you explain this?	
(C)	You indicated changed adherence/engagement patterns in (E1-E3) subgroups. How would you explain these patterns?
**Probes**:	Cultural issues? Have you managed to address this reduced engagement?
	Have you had clients who have agreed to tele-MH, and then had to pull out for some reason? What were the reasons?

4. **Access/acceptance by MH providers**

**Table:**

1.	Overall, how do you think MH providers have felt/dealt with the implementation/switch?
**Probes**:	What have they expressed difficulties with? How did you overcome these difficulties?
	Do their difficulties differ by intervention type?
	Did their views and/or difficulties change over time?

5. **MHAM perspective**

**Table:**

1.	You indicated that you find tele-MH (F1) as an alternative to face-to-face counselling. Can you explain this choice to me?
**Probes**:	Do you think it is more useful for a given “intervention” type?
	Was there a specific “intervention” where tele-MH was the most difficult to implement? Why?
	How do you think it can be made more useful? Is there any specific resources or support that could be provided?
	What other alternatives do you think could/should be explored by MSF?

**Part 5—Advantages**

**Table:**

1.	You indicated (G1) as advantages of tele-MH compared to face-to-face sessions. Can you expand on this for me?
**Probes**:	Do these advantages differ by intervention type, or by subpopulation? Why do you think this is?

**Part 6—Experiences where tele-MH implementation not possible**

I would like to ask you about your experiences with interventions that could not be adapted to tele-MH (clarify with responses given to introduction question 1).

**Table:**

1.	What aspects of the intervention made you decide that the switch was not possible?
**Probes**: Is there anything that could have helped you to overcome these challenges? Can you describe what would have helped?	
2.	Were you able to offer any other solution for this MH intervention, other than tele-MH?
**Probes**: If yes, can you describe the solution for me?	

**Part 7—Experiences with programmes where tele-MH implementation was not possible**

I would like to ask you questions about programmes where the switch to tele-MH was not possible at all.

**Table:**

1.	You identified that you were not able to adapt the (B1 comment) programme to tele-MH. Can you tell me a bit about why you were not able to adapt to this programme?
**Probes:**	challenges mainly related to the local context? Project/operations? Patients?
	Is there anything that could have helped you to overcome these challenges? IF so, what?
2.	Were you able to offer any other solution for this programme, other than tele-MH?
**Probes**:	If yes, can you describe the solution for me?

**Part 8—Conclusion**

**Table:**

1.	What advice would you give to others starting the switch?
2.	Do you think your experiences could change the way mental health activities are implemented in the future?
**Probes**:	How so?
3.	Is there anything that you feel that we may have missed that you think is important for us to know/consider?
4.	Do you have any questions for us?

Thank you for your time.

## References

[1] Saxena S, Thornicroft G, Knapp M, Whiteford H (2007). Resources for mental health: scarcity, inequity, and inefficiency. Lancet.

[2] Augusterfer EF, O'Neal CR, Martin SW, Sheikh TL, Mollica RF (2020). The Role of telemental health, tele-consultation, and tele-supervision in post-disaster and low-resource settings. Curr Psychiatry Rep.

[3] Acharibasam JW, Wynn R (2018). Telemental health in low- and middle-income countries: a systematic review. Int J Telemed Appl.

[4] De Luca R, Calabro RS (2020). How the COVID-19 pandemic is changing mental health disease management: the growing need of telecounselling in Italy. Innov Clin Neurosci.

[5] Stevens GJ, Hammond TE, Brownhill S (2019). SMS SOS: a randomized controlled trial to reduce self-harm and suicide attempts using SMS text messaging. BMC Psychiatry.

[6] Hilty DM, Cobb HC, Neufeld JD, Bourgeois JA, Yellowlees PM (2008). Telepsychiatry reduces geographic physician disparity in rural settings, but is it financially feasible because of reimbursement?. Psychiatr Clin North Am.

[7] Carter H, Araya R, Anjur K, Deng D, Naslund JA (2021). The emergence of digital mental health in low-income and middle-income countries: a review of recent advances and implications for the treatment and prevention of mental disorders. J Psychiatr Res.

[8] Pahuja E, Kumar S, Kumar A (2020). Collaborative video consultations from tertiary care based telepsychiatrist to a remote primary care doctor to manage opioid substitution therapy clinic. J Neurosci Rural Pract.

[9] Sivakumar PT, Mukku SSR, Kar N (2020). Geriatric telepsychiatry: promoting access to geriatric mental health care beyond the physical barriers. Indian J Psychol Med.

[10] Iqbal Y, Jahan R, Yesmin S, Selim A, Siddique SN (2020). COVID-19-related issues on tele-counselling helpline in Bangladesh. Asia Pac Psychiatry.

[11] Green AS, Ruchman SG, Katz CL, Singer EK (2020). Piloting forensic tele-mental health evaluations of asylum seekers. Psychiatry Res.

[12] Adepoju P (2020). Africa turns to telemedicine to close mental health gap. Lancet Digit Health.

[13] Bradford NK, Caffery LJ, Smith AC (2016). Telehealth services in rural and remote Australia: a systematic review of models of care and factors influencing success and sustainability. Rural Remote Health.

[14] Brainard JS, Ford JA, Steel N, Jones AP (2016). A systematic review of health service interventions to reduce use of unplanned health care in rural areas. J Eval Clin Pract.

[15] Caffery LJ, Farjian M, Smith AC (2016). Telehealth interventions for reducing waiting lists and waiting times for specialist outpatient services: a scoping review. J Telemed Telecare.

[16] Guinart D, Marcy P, Hauser M, Dwyer M, Kane JM (2021). Mental health care providers' attitudes toward telepsychiatry: a systemwide, multisite survey during the COVID-19 pandemic. Psychiatr Serv.

[17] Chakrabarti S (2015). Usefulness of telepsychiatry: a critical evaluation of videoconferencing-based approaches. World J Psychiatry.

[18] Chi NC, Demiris G (2015). A systematic review of telehealth tools and interventions to support family caregivers. J Telemed Telecare.

[19] Fu Z, Burger H, Arjadi R, Bockting CLH (2020). Effectiveness of digital psychological interventions for mental health problems in low-income and middle-income countries: a systematic review and meta-analysis. Lancet Psychiatry.

[20] Yang Y, Li W, Zhang Q, Zhang L, Cheung T, Xiang YT (2020). Mental health services for older adults in China during the COVID-19 outbreak. Lancet Psychiatry.

[21] Aitken M, de St Jorre J, Pagliari C, Jepson R, Cunningham-Burley S (2016). Public responses to the sharing and linkage of health data for research purposes: a systematic review and thematic synthesis of qualitative studies. BMC Med Ethics.

[22] Martinez-Martin N, Dasgupta I, Carter A (2020). Ethics of digital mental health during COVID-19: crisis and opportunities. JMIR Ment Health..

[23] Creswell JW, Plano Clark VL, Gutmann ML, Hanson WE, Tashakkori A, Teddlie C (2003). Advance mixed methods research designs. Handbook of mixed methods in social and behavioral research.

[24] Mucic D (2010). Transcultural telepsychiatry and its impact on patient satisfaction. J Telemed Telecare.

[25] Chipps J, Brysiewicz P, Mars M (2012). Effectiveness and feasibility of telepsychiatry in resource constrained environments? A systematic review of the evidence. Afr J Psychiatry (Johannesbg).

[26] Myers KM, Vander Stoep A, McCarty CA, Klein JB, Palmer NB, Geyer JR, Melzer SM (2010). Child and adolescent telepsychiatry: variations in utilization, referral patterns and practice trends. J Telemed Telecare.

[27] Joint Task Force for the Development of Telepsychology Guidelines for Psychologists (2013). Guidelines for the practice of telepsychology. Am Psychol.

[28] Hilty DM, Gentry MT, McKean AJ, Cowan KE, Lim RF, Lu FG (2020). Telehealth for rural diverse populations: telebehavioral and cultural competencies, clinical outcomes and administrative approaches. Mhealth.

[29] Smith K, Ostinelli E, Macdonald O, Cipriani A (2020). COVID-19 and telepsychiatry: development of evidence-based guidance for clinicians. JMIR Ment Health.

